# Serum macroprolactin levels in pregnancy and association with thyroid autoimmunity

**DOI:** 10.1186/s12902-015-0025-2

**Published:** 2015-06-20

**Authors:** Metin Guclu, Soner Cander, Sinem Kiyici, Ebru Vatansever, Arif Bayram Hacihasanoğlu, Gurcan Kisakol

**Affiliations:** Department of Endocrinology and Metabolism, Sevket Yilmaz Research and Education Hospital, Bursa, Turkey; Department of Internal Medicine, Sevket Yilmaz Research and Education Hospital, Bursa, Turkey; Department of Biochemistry, Sevket Yilmaz Research and Education Hospital, Bursa, Turkey

**Keywords:** Pregnancy, Prolactin, Macroprolactin, Thyroid dysfunction, Thyroid autoimmunity

## Abstract

**Bacground:**

To assess the contribution of macroprolactin to high serum prolactin levels and their association with thyroid status and thyroid autoimmunity during pregnancy.

**Methods:**

138 pregnant women who suspected of having thyroid dysfunction were studied and divided into three groups according to the thyroid status; group 1; euthyroidism (n 40), group 2; hypothyroidism (n 54), and group 3; hyperthyroid (n 44). Polyethylene glycol (PEG) precipitation method was used for detection of macroprolactin. A percentage recovery of 40 % or less is considered as macroprolactinemia. If macroprolactin was negative, the percentage of monomeric prolactin recovery (monoPRL %) after PEG precipitation was used for comparison between the groups.

**Results:**

Macroprolactinemia was found in two patients (1.4 %) one from hypothyroid and other from euthyroid group. Basal prolactin levels in these patients were 400 and 403 ng/mL respectively. Referring to all patients, there was no correlation between PRL, macroPRL or monoPRL % with thyroid hormone status and also with the serum levels of thyroid antibodies (p > 0.05). A positive correlation was observed between the serum levels of PRL with TSH (p = 0.014 and r = 0.219), while a negative correlation was found with FT4 (p = 0.011 and r = −0.227).

**Conclusions:**

Despite the fact that serum prolactin levels were found to be high during pregnancy, the contribution of macroprolactin was found to be insignificant in our study. Unlike other auto immune diseases, we could not find any relationship between thyroid autoimmunity and PRL, macroPRL or monoPRL %. These results confirmed that measured prolactin was quite homogeneous during pregnancy.

## Background

Prolactin, a protein hormone, is secreted from the anterior pituitary usually in response to physiological and rarely pathological stimuli. Prolactin concentrations begin to increase after 6 weeks of pregnancy and reach the highest level in late pregnancy to prepare the mammary glands of the breasts for the production of milk [1–3]. It is well known that circulating prolactin is not homogenous and three different forms are defined according to molecular size: monomeric PRL (monoPRL), big PRL (bigPRL), and macroprolactin (macroPRL). Macroprolactin is described as a complex of PRL with immunoglobulin G (IgG) which is related to antiprolactin autoantibodies. This formulation of PRL may cause limited bioactivity, bioavailability and clearance from the glomerulus. Macroprolactinemia is usually asymptomatic, patients with macroprolactinemia do not need further therapy, and pregnancy is possible without any treatment for such hyperprolactinemia [4–6]. Although the gold standard method for detecting macroPRL is gel filtration chromatography, precipitation with polyethylene glycol (PEG) is a widely used screening test. A low PRL recovery, <40 %, after PEG precipitation indicates the presence of macroPRL [7, 8]. In hyperprolactinemic states, the contribution of macroprolactin has been found to be 10 to 25 % of prolactin and macroprolactinemia may be present in about 0.2–3.6 % of the general population [9].

Normal maternal thyroid function is essential for both fetal and maternal health and many physiological alterations occur during pregnancy. Human chorionic gonadotropin (hCG) and estrogen are two pregnancy-related hormones responsible for increased thyroid hormone levels in the blood. Thyroid disorders, particularly those with autoimmune origin, are common in women of reproductive age and both hypo and hyperthyroidism were reported to be associated with poor pregnancy outcome [10–12]. Therefore, different trimester-specific reference ranges were defined to distinguish physiological alterations from pathological states [13–15]. It has been shown that the thyrotropin-releasing hormone (TRH) can stimulate the secretion of prolactin in experimental conditions; however its physiological influence is not so obvious. Hypothyroidism may cause pituitary and thyrotrophic hyperplasia which result in elevated TRH and prolactin release. The high levels of both hormones may return to normal after effective thyroid hormone replacement therapy [16, 17].

Previous studies have shown that prolactin also plays an essential role in metabolism and regulation of the immune system and macroprolactin has been described as the major immune reactive prolactin species in the serum of several subjects before and during pregnancy. The relationship with anti-PRL autoantibodies and autoimmune disorders has been investigated in some studies due to the structure of macroprolactin [18, 19]. Although some case reports have shown interaction between macroprolactinemia and autoimmune thyroid disorders, other studies examining a large number of patients revealed no specific association. It has been shown that only a minority of patients with SLE had higher frequencies of macroprolactinemia [20, 21]. The association of prolactin and macroprolactin with thyroid hormones and thyroid autoantibodies during pregnancy has not been studied before.

In the present study, we aimed to assess the contribution of macroprolactin to high serum prolactin levels and the association of prolactin (PRL) and macroprolactin (macroPRL) levels with thyroid status and thyroid autoimmunity during pregnancy.

## Methods

The study, designed as a prospective case series, was conducted between March 2014 and December 2014 in the Sevket Yılmaz Research and Education Hospital, Bursa, Turkey. Data were collected by selecting pregnant women who had undergone a TSH screening and been directed to endocrinology consultation. The study protocol was approved by the local ethics committee. A total of 138 pregnant women suspected of having thyroid dysfunction, and who gave informed consent, were studied in different stages of gestation. All subjects were interviewed regarding reproductive history including miscarriages, preterm deliveries, and neonatal complications. Personal histories of thyroid disorders and thyroid medications were noted. Selected patients were divided into three groups according to the ATA guidelines; Group 1 euthyroid (TSH between 0.1 μIU/L and 2.5 μIU/L), group 2 hypothyroid (TSH ≥ 2.51 μIU/L), and group 3 hyperthyroid (TSH ≤ 0.09 μIU/L). The TPOAb ≥ 35 U/mL was considered to be antibody positive also according to ATA guidelines [15]. History of the following complications during previous pregnancies were considered to be complicated pregnancy; miscarriage, stillbirth, preterm birth, fetal death, postpartum hemorrhage, placental abruption, cardiac dysfunction and maternal heart failure, gestational hypertension, preeclampsia, gestational diabetes, and cesarean sections.

### Laboratory investigation

Following an overnight fast, blood samples were acquired in the morning from each subject. Serum was isolated by centrifugation and stored at −80 °C until testing, if needed. The concentrations of serum TSH, FT3 and FT4 were detected by electrochemiluminescence immunoassay diagnostic kit (Advia Centaur XP Siemens, USA). TGAb and TPOAb levels were detected using an electrochemiluminescence immunoassay diagnostic kit (Roche Diagnostics Ltd., Basel, Switzerland). The concentration of serum prolactin was detected by electrochemiluminescence immunoassay diagnostic kit (Immulite 2000, Diagnostic Products Corporation, LA, CA). After centrifugation, the supernatant containing the unprecipitated prolactin is tested for total prolactin level. A concentration of 25 % PEG is added to an aliquot of serum specimen and the PEG-treated sample is incubated for a short period and then centrifuged to precipitate out macroprolactin. The supernatant containing the unprecipitated prolactin is tested for recovery of basal prolactin. The PEG-precipitable PRL (%), which represents the amount of macroprolactin, is calculated as follows: (total PRL-free PRL)/total PRL *×* 100. PEG-precipitation ratio greater than 60 % (recovery less than 40 %) is used as the cut-off value for the diagnosis of macroprolactinemia. Recovery of less than 40 % after PEG is considered a diagnostic criteria for macroprolactin; recovery values around 40–60 % are considered borderline, and values >60 % rule out macroprolactinemia [22–24]. The unprecipitated percentage of basal prolactin is considered as recovery of monomeric prolactin.

### Statistical analysis

Statistical analysis was performed using IBM-SPSS software (version 21.0; SPSS Inc., Chicago, IL, USA). Comparisons between groups were performed using ANOVA for normally distributed variables, and the Mann–Whitney and Kruskal-Wallis tests for non-normal variables. All data are expressed as mean ± standard deviation or percentages, while p value <0.05 was considered statistically significant.

## Results

Comparison of patients’ demographics and laboratory parameters between the groups are shown in Table 1.

**Table 1:** Comparison of demographic and laboratory parameters between the groups

Data	Group 1	Group 2	Group 3	*p*
	Euthyroid	Hypothyroid	Hyperthyroid	
	(n = 40)	(n = 54)	(n = 44)	
Age (years)	29.2 ± 5.6	29.0 ± 5.1	29.3 ± 4.9	NS
Mean number of pregnancies	2.2	2.4	2.7	NS
Number of patients with history of complicated pregnancy (n)	12	18	14	NS
Weight (kg)	69.9 ± 13.9	68.7 ± 14.5	60.7 ± 11.5^a,b^	<0.005
SBP (mmHg)	111.5 ± 12.5	111.7 ± 11.9	130.5 ± 13.7	NS
DBP (mmHg)	73.2 ± 8.9	72.3 ± 9.1	71.4 ± 10.7	NS
TSH(μIU/L)	1.04 ± 0.7	6.7 ± 10.7^a,c^	0.02 ± 0.02	<0.001
FT4(ng/dL)	1.13 ± 0.21	1.17 ± 0.54	1.7 ± 0.80^a,b^	<0.001
FT3 (pg/mL)	2.93 ± 0.33	2.78 ± 0.53	4.84 ± 2.04^a,b^	<0.001
TPOAb (U/mL)	189.9 ± 352.6	563.8 ± 576.5^c^	158.8 ± 308.8	<0.001
TGAb(U/mL)	71.1 ± 111.5	164.7 ± 159.0^c^	123.7 ± 311.2	<0.05
PRL(ng/mL)	57.4 ± 49.4	69.6 ± 74.8	54.7 ± 68.7	NS
macroPRL (n)	1	1	-	NA
monoPRL %	90.6 ± 8.7	91.5 ± 9.2	88.9 ± 10.9	NS

*SBP* systolic blood pressure, *DBP* diastolic blood pressure, *TSH* thyroid stimulating hormone. *FT4* free tetraiodotronin, *FT3* free triiodotronin, *TPOAb* thyroid peroxidase antibody, *TGAb* thyroglobulin antibody, *PRL* prolactin. monoPRL% the percentage of monomeric prolactin recovery after PEG precipitation

a = group 3 vs group2 p < 0.05; b = group 3 vs group 1 p < 0.001. c = group 2 vs group 1 p < 0.00

The number of patients in the euthyroid (n = 40), hypothyroid (n = 54) and hyperthyroid (n = 44) groups and age distributions between the groups (mean age was 29.2 ± 5.6 years in group 1, 29.0 ± 5.1 years in group 2 and 29.3 ± 4.9 years in group 3) were similar (p > 0.05). Although it did not reach statistical significance, the mean number of previous pregnancies was higher in hyperthyroid patients (2.7) than hypothyroid (2.4) and euthyroid (2.2) patients. Rates of complicated pregnancy history were high in all groups (n = 12 (30 %) in group 1, n = 18 (33 %) in group 2 and 14 (34 %) in group 3).

Body weight of the patients in group 3 was lower than patients in the other groups (p < 0.005) and the mean weight was 69.9 ± 13.9 kg in group 1, 68.7 ± 14.5 kg in group 2 and 60.7 ± 11.5 kg in group 3. Blood pressure measurements were not different between the groups. Mean TSH level was 1.04 ± 0.7 μIU/L in group 1, 6.7 ± 10.7 μIU/L in group 2, and 0.02 ± 0.02 μIU/L in group 3. Mean FT4 was 1.13 ± 0.21 ng/dL in group 1, 1.17 ± 0.54 ng/dL in group 2, and 1.7 ± 0.80 ng/dL in group 3. Mean FT3 pg/mL was 2.93 ± 0.331 pg/mL in group 1, 2.78 ± 0.531 pg/mL in group 2, and 4.84 ± 2.04 pg/mL in group 3. As expected, there were significant differences in serum TSH, FT4, and FT3 levels between the groups (p < 0.001). There were significant differences also in terms of thyroid antibodies between the groups (p < 0.001). TPOAb was 189.9 ± 352.6 U/mL in group 1, 563.8 ± 576.5 U/mL in group 2, and 158.8 ± 308.8 U/mL in group 3. TGAb was 71.1 ± 111.51 U/mL in group 1, 164.7 ± 159.0 U/mL in group 2, and 123.7 ± 311.2 U/mL in group 3. Group 2 had significantly higher TPOAb and TGAb levels compared to group 1 and 3 (p < 0.01 and <0.05 respectively). Macroprolactinemia was detected in two patients. One patient was from group 1 and the other patient was from group 2. Basal PRL levels of these patients were 400 and 403 ng/mL respectively. Basal serum PRL levels and percentage of monoPRL recovery after PEG precipitation were similar when the three groups were compared. Percentages of monoPRL recovery after PEG treatment were not different between the groups; 90.6 ± 8.7 % in group 1, 91.5 ± 9.2 % in group 2, and 88.9 ± 10.9 % in group 3.

Correlation analyses of prolactin and monoPRL % with patients’ characteristics and laboratory parameters are summarized in Table 2**.**

**Table 2:** Correlation analyses of prolactin and monoPRL % with patient’s demographics and laboratory parameters

Data	Prolactin		monoPRL %	
	*p* value	r	*p* value	r
Age (years)	0.635	−0.043	0.282	−0.096
Gestation week	<0.001	0.532	0.114	−0.144
Number of pregnancies	0.001	−0.296	0.699	0.035
History of complicated pregnancy	0.176	−0.123	0.918	0.009
Weight	0.271	0.099	0.457	−0.067
TSH	0.014	0.219	0.341	0.086
FT4	0.011	−0.227	0.982	−0.002
FT3	0.051	−0.174	0.706	−0.034
TPOAb	0.581	−0050	0.980	−0.002
TGAb	0.766	−0.031	0.393	0.089
PRL	-	-	0.043	−0.181
monoPRL %	0.043	−0.181	-	-

*TSH* thyroid stimulating hormone, *FT4* free tetraiodotronin, *FT3* free triiodotronin, *TPOAb* thyroid peroxidase antibody, *TGAb* thyroglobulin antibody, *PRL* prolactin, monoPRL% percentage of monomeric prolactin recovery after PEG precipitation

There was a positive correlation (p < 0.001, r =0.532) between PRL with gestational week and a negative correlation (p < 0.05, r = −0.296) with number of pregnancies. Serum prolactin levels were also found positively correlated with serum TSH (p = 0.014 and r = 0.219) and negatively correlated with FT4 (p = 0.011 and r = −0.227). Because of the insufficient number of macroprolactin positive patients, correlation analyses were done between the percentages of monoPRL recovery instead of macroprolactinemia. However, no statistically significant correlation was found between monoPRL % and thyroid hormone levels. Also, there was no correlation between the serum levels of thyroid antibodies with PRL and monoPRL % (p > 0.05). There was a slightly significant negative correlation between PRL and monoPRL % (p = 0.043 and r = −0.181).

The comparisons of data obtained from patients who were divided into two groups according to the presence of TPOAb antibody are also seen in Table 3.

**Table 3:** Comparisons of PRL and monoPRL% between patient with and without history of complicated pregnancy and TPOAb positivity

Parameter	TPOAb		p
	Positive	Negative	
	(n 81)	(n 57)	
PRL(ng/mL)	66.28 ± 73.8	53.2 ± 51.3	0.923
monoPRL %	90.73 ± 9.53	90.14 ± 10.0	0.666
TSH(μIU/L)	3.97 ± 9.3	1.35 ± 0.62	0.003
FT4(ng/dL)	1.28 ± 0.65	1.46 + 0.62	0.018
FT3 (pg/mL)	3.36 + 1.61	3.68 + 1.39	0.079

*TSH* thyroid stimulating hormone, *FT4* free tetraiodotronin, *FT3* free triiodotronin, *TPOAb* thyroid peroxidase antibody, *PRL* prolactin, monoPRL% percentage of monomeric prolactin recovery after PEG precipitation

Patients with positive TPOAb had higher serum TSH levels (3.97 ± 9.3 vs. 1.35 ± 0.62 μIU/L) and lower serum FT4 levels (1.28 ± 0.65 vs. 1.46 + 0.62 ng/dL) compared to patients with negative TPOAb and the differences between the groups were significant for both parameters (p = 0.003 and 0.018 respectively). But serum FT3 levels of the groups were similar. In addition, neither prolactin nor monoPRL % levels were different between the patients with positive and negative TPOAb (p > 0.05).

Comparisons of PRL, monoPRL %, and thyroid parameters between patients with and without a history of complicated pregnancy are shown in Table 4.

**Table 4:** Comparisons of PRL, monoPRL %, and thyroid parameters between patient with and without history of complicated pregnancy

Parameter	History of complicated pregnancy		p
	Positive	Negative	
	(n 44)	(n 94)	
PRL (ng/mL)	49.12 ± 66.0	66.64 ± 65.9	0.026
monoPRL %	90.7 ± 11.1	90.50 ± 9.02	0.788
TSH(μIU/L)	2.72 ± 4.01	3.06 ± 8.5	0.655
FT4(ng/dL)	1.28 ± 0.49	1.37 + 1.67	0.305
FT3 (pg/mL)	3.32 + 1.14	3.59 + 1.67	0.811
TPOAb (U/mL)	344.7 ± 496.9	207.7 ± 468.8	0.045
TGAb(U/mL)	169.3 ± 329.2	89.9 ± 134.7	0.026

*TSH* thyroid stimulating hormone, *FT4* free tetraiodotronin, *FT3* free triiodotronin, *TPOAb* thyroid peroxidase antibody, *PRL* prolactin, monoPRL% percentage of monomeric prolactin recovery after PEG precipitation

In terms of history of complicated pregnancy, there was a slightly significant difference for PRL levels between the groups. Patients without a history of complicated pregnancy had higher prolactin levels than the patients with a history of complicated pregnancy (66.64 ± 65.9 vs. 49.12 ± 66.0 ng/ml; p = 0.026). There were also significant differences in terms of thyroid autoantibodies between the two groups. Patients with a history of complicated pregnancy had higher antibody levels. Serum TPOAb and TGAb levels were 344.7 ± 496.9 U/mL and 169.3 ± 329.2 U/mL in patients with complicated pregnancy, whereas they were 207.7 ± 468.8 U/mL and 89.9 ± 134.7 U/mL in patients without complicated pregnancy.

## Discussion

Pregnancy and breastfeeding are the most important periods of long-term physiological increase of prolactin levels. Absence of galactorrhea is mostly related with the high levels of estrogen during pregnancy despite hyperprolactinemia [25]. On the other hand, a probable alternative explanation for the absence of galactorrhea may be heterogeneity of prolactin molecules. It is well known that high-molecular weight isoforms of PRL have low binding affinity to PRL receptors. In addition, it was shown that the relative proportion of little PRL increased in pregnant women as pregnancy progressed [1]. In some studies, the presence of big, big PRL has been reported in a range of 8–38 % of total PRL during pregnancy [26, 27]. However, Hattori et al. [28] has found that the frequency of macroprolactinemia was 2.9 % in women during the third trimester of pregnancy (3 of 105). We found the frequency of macroprolactinemia as 1.4 % in women in the first and second trimester. Unlike the other studies [26, 27], our results were similar to the results of Hattori et al. [28]. The small number of patients with macroprolactinemia suggested that the increase of prolactin is quite homogenous during pregnancy. Interestingly, regardless of thyroid status, patients with macroprolactinemia had the highest PRL levels (400 and 403 ng/mL) compared with other patients. Our results were also similar to Pascoe-Lira et al.’s study. They found that 3.8 % of the pregnant women had significant macroprolactinemia and total PRL concentration was observed higher in those patients. Another probable explanation for the discrepancy of published results may be the detection method which is commonly used for macroprolactin measurement. Although the gold standard method for detecting prolactin homogeneity or macroPRL is gel filtration chromatography, precipitation with polyethylene glycol (PEG) is a widely used, inexpensive, and easy screening test [7, 8, 29]. Therefore, if we were using the latter method, results could have been different. In general, however, this method is not widely used, or available everywhere and recommended if borderline measurement was obtained. In other words, gel filtration chromatography would be necessary if 40-60 % of prolactin recovery was detected after PEG precipitation [30]. Almost all of our patients have shown >90 % persistency from initial levels of monomeric prolactin after PEG precipitation despite the clinical and laboratory differences. These data have supported the strong homogeneity for PRL during pregnancy as well.

It has been shown that there is a strong association between thyroid and prolactin secretion physiologically. Many critical and period-specific changes occur during pregnancy in thyroid and prolactin metabolism. Due to the structural similarity, elevated hCG may increase the synthesis of thyroid hormones and cause TSH suppression. Thus PRL response to TRH may be blunted in hyperthyroid conditions. In contrast, elevated TRH can increase levels of TSH and PRL in patients with hypothyroidism [31, 32]. Autoimmune thyroid disorders are common in women of reproductive age and subclinical hypothyroidism related to chronic autoimmune thyroiditis is the most common thyroid disorder. If the classic reference range (0.4–4.0 μIU/L) of TSH is used in the first trimester of pregnancy, subclinical cases of hypothyroidism may be missed. Gestational transient thyrotoxicosis (GTT) or hCG-mediated transient TSH suppression and Graves Disease (GD) are the most common types of hyperthyroid disease in pregnant women. GTT is generally asymptomatic with mild biochemical hyperthyroidism and may be exacerbated by hyperemesis, while GD is a real thyrotoxic disease and mostly requires anti-thyroid treatment. Both hypo and hyperthyroidism were reported to be associated with poor pregnancy outcome. These outcomes include spontaneous abortions, preterm birth, intrauterine growth restriction, stillbirth, preeclampsia, heart failure, and neurodevelopment deficits in the children [10–12].

In the present study, due to the complex relationship between thyroid and prolactin metabolism, we tried to examine patients by dividing them into three groups in terms of thyroid status. The second aim of our study was to evaluate prolactin homogeneity in different thyroid disorders during pregnancy. To the best of our knowledge, the present study is the first study which investigated this relationship. Contrary to our expectations, macroprolactin levels did not increase during pregnancy and there were only two patients with macroprolactinemia. All of the remaining patients showed homogeneity in terms of prolactin size. We preferred to use percentage of monoPRL recovery for the other patients to define homogeneity instead of macroprolactinemia for two reasons. Firstly because of the insufficient number of patients with macroprolactinemia and the secondly due to the fact that the definition of macroprolactinemia is already based on percentage of recovered prolactin after PEG precipitation. Nevertheless, there was a small amount of data that can be considered significant. These findings were as follows, a slightly significant positive correlation between TSH and PRL, and a negative correlation between FT4 and PRL. Although there is a known close relationship between thyroid and prolactin metabolism, it was not so clear in the present study. However, the number of marked hypothyroid and hyperthyroid patients was inconsiderable in our study and most of our patients were euthyroid. This can explain why we found serum prolactin levels similar and not enough to confirm our hypothesis.

The other potential issue is the relationship of prolactin with autoimmune processes. It was shown that prolactin has the potential to stimulate the immune system and can also be produced in extra pituitary sites [33, 34]. A large number of autoantibodies are increased in patients with hyperprolactinemia without overt clinical autoimmune disease. These antibodies include antipituitary, antithyroid peroxidase, antithyroglobulin, anti gastric parietal cells, antimicrosomal, and anticardiolipin antibodies [35–38]. In addition, there is an intriguing relationship between macroprolactin and some collagen tissue disorders such as SLE. It is proposed that there is a correlation between disease activity and macroprolactinemia [39, 40]. Initially we investigated this relationship in different thyroid hormone status. We could not find any relationship between thyroid antibodies with either PRL or monoPRL %. Regardless of thyroid status, according to the presence of TPOAb alone, the negative tendency continued. It is well known that antibodies may be high in healthy individuals without apparent thyroid disease [41, 42]. Presence of high number of patients with positive thyroid antibody, 81(58 %) in all groups, can be a reason for statistical uncertainty.

## Conclusions

In conclusion; there were no significant contributions of macroprolactin to prolactin levels during pregnancy. The levels of prolactin showed strong homogeneity and no correlation were found with thyroid dysfunction and thyroid autoimmunity at that time. This uncertainty persisted when correlation analyses was done with monoPRL % instead of macroPRL. Ultimately, we could not find any relationship between thyroid hormones and antibodies with prolactin types in terms of molecular size.

### Ethical approval

All procedures performed in studies involving human participants were in accordance with the ethical standards of the institutional and/or national research committee and with the 1964 Helsinki declaration and its later amendments or comparable ethical standards.

The study protocol was approved by Sevket Yilmaz Research and Education Hospital Ethics Committee. (Number and date: 2014/0501-07.03.2014).

### Informed consent

Informed consent was obtained from all individual participants included in the study.

## Abbreviations

DBP: Diastolic blood pressure
FT3: Free triiodotronin
FT4: Free tetraiodotronin
macroPRL: Macroprolactin
monoPRL %: the percentage of monomeric prolactin recovery after PEG precipitation
PRL: Prolactin
PEG: Polyethylene glycol
SBP: Systolic blood pressure
TGAb: Thyroglobulin antibody
TPOAb: Thyroid peroxidase antibody
TSH: Thyroid stimulating hormone
